# *Wolbachia* Impacts *Anaplasma* Infection in *Ixodes scapularis* Tick Cells

**DOI:** 10.3390/ijerph19031051

**Published:** 2022-01-18

**Authors:** Kalin M. Skinner, Jacob Underwood, Arnab Ghosh, Adela S. Oliva Chavez, Corey L. Brelsfoard

**Affiliations:** 1 Department of Biological Sciences, Texas Tech University, 2901 Main St., Lubbock, TX 79409, USA; kalin.skinner@ttu.edu (K.M.S.); leo1985.arnab@gmail.com (A.G.); 2 Department of Entomology, Texas A & M University, 370 Olsen Blvd, College Station, TX 77843, USA; jund8841@gmail.com (J.U.); aolivachavez@tamu.edu (A.S.O.C.)

**Keywords:** transfection, endosymbiont, tick, virus inhibition, *Wolbachia*

## Abstract

The specific interactions of members of tick bacterial microbiota and their effects on pathogen transmission remains relatively unexplored. Here, we introduced a novel *Wolbachia* infection type into *Ixodes scapularis* tick cells and examined the antipathogenic effects on the intracellular pathogen *Anaplasma phagocytophilum*. An increase in *A. phagocytophilum* replication was observed in *Wolbachia*-infected tick cells. However, *Wolbachia* infection densities decreased when cells were serially passaged and ultimately the infection was lost. Host-cell immune response was also examined as an additional factor that could have affected *A. phagocytophilum* replication in *Wolbachia*-infected cells. In early passages post-*Wolbachia* infection, a decreased immune response was observed, but in later passages of cells with low *Wolbachia* densities, there was no change in the immune response. The results are discussed in relation to the importance of studying the interactions of the tick microbiota, the host cell, and the pathogen and the development of novel tick and tick-borne disease-control approaches.

## 1. Introduction

Ticks are a noteworthy vector of multiple human pathogens that result in diseases, including Lyme disease, babesiosis, anaplasmosis, rocky mountain spotted tick fever, southern tick-associated rash illness (STARI), and ehrlichiosis [1,2]. The composition of native microbiota of ticks has been demonstrated to profoundly influence many aspects of tick biology, including the ability of ticks to transmit pathogens [3,4,5]. Members of the bacterial microbiota include symbionts from several genera including: *Lariskella*, *Arsenophornus*, *Sprioplasma*, *Rickettsia*, *Rickettsiella*, *Francisella*, *Midichloria*, *Coxiella*, *Cardinium*, and *Wolbachia* [2,6,7,8]. Furthermore, we are beginning to appreciate that pathogen infection can alter the bacterial microbiota of ticks [4,6]. However, little is known about the specific interactions of members of the bacterial microbiota of ticks and their impact on disease transmission [9,10].

Naturally occurring *Wolbachia* infections have been reported in multiple tick species, including *Ixodes scapularis*, *Ixodes ricincus*, *Rhipicephalus sanguineus*, and *Amblyomma americanum*, to name a few [2,9,11,12]. *Wolbachia* has received particular attention in the context of mosquito-borne vector control for its ability to alter host fitness and interfere with pathogen transmission via arthropod vectors. *Wolbachia* is an obligate intracellular bacterium found in >55% of insects, in addition to terrestrial crustaceans, arachnids, filarial nematodes, and acari [13,14]. *Wolbachia* in arthropods causes alterations in host reproduction, resulting in phenotypes such as feminization, male killing, parthenogenesis and cytoplasmic incompatibility (CI) [13]. Because *Wolbachia*-infected females can mate and produce viable offspring with infected and uninfected males, they are afforded a reproductive advantage which can drive a given disease refractory phenotype into a natural population [15,16]. An additional important feature of *Wolbachia* is its ability to induce resistance to various pathogens in its insect hosts [17,18,19]. Recent studies show that *Wolbachia* induces production of reactive-oxygen species (ROS), which then activate the Toll and Imd innate immunity pathways to induce the expression of antiviral effectors [20]. In the *Drosophila* host, native *Wolbachia* can also confer resistance to viral pathogens [21]. *Wolbachia* density-dependent inhibition of dengue virus replication has also been observed in mosquito cell lines [22,23]. On the other hand, in the mosquito *Culex tarsalis*, females showed an increase in West Nile Virus titers when injected with *Wolbachia*, and *Wolbachia*-infected *Anopheles stephensi* show limited protection against *plasmodium* infection [24], suggesting *Wolbachia* effects cannot be generalized but may be specific to the host, pathogen, and *Wolbachia* strain type. While *Wolbachia* infections have been successfully established in tick cell lines [12], no work has been performed to investigate the effect of *Wolbachia* on tick-vectored pathogens.

Here, we introduced a novel *Wolbachia* infection type into *I. scapularis* tick cells and examined for antipathogenic effects on the intracellular pathogen *Anaplasma phagocytophilum. Wolbachia* infection densities decreased when cells were serially passaged, and the introduced infection was subsequently lost. An increase in *A. phagocytophilum* replication was also observed in *Wolbachia* infected ISE6 cells. The results are discussed in the context of previous *Wolbachia* transfection experiments and pathogen inhibitory effects observed in other arthropod systems.

## 2. Materials and Methods

### 2.1. ISE6 Cell Transfection

*Aedes albopictus* Aa23 cells infected with the *w*albB *Wolbachia* type were grown to >85–95% confluency in Schneider’s insect medium (SM) (Milipore Sigma, St. Louis, MO, USA) supplemented with heat-inactivated 10% fetal bovine serum (FBS) in 75 cm^2^ cell culture flasks (TPP—Techno Plastic Products, Trasadingen, Switzerland). Extracellular *w*albB was isolated from Aa23 cells using a modified procedure as previously described [25]. Approximately 500 µL of the extracellular *Wolbachia* extract was added to SM with 10% heat-inactivated FBS in a 25 cm^2^ flask and incubated at 28 °C to examine for environmental bacteria and/or fungal contamination before inoculating naïve host cells.

*Ixodes scapularis* ISE6 cells were obtained from Dr. Timothy Kurtti at the University of Minnesota. ISE6 cells were cultured in L-15 B media supplemented with 10% Tryptone Phosphate Broth, 0.1% Lipoprotein concentrate, 2 mM L-glutamate, and 5% heat inactivated FBS in 25 cm^2^ flasks (TPP—Techno Plastic Products, Trasadingen, Switzerland) at 28 °C at atmospheric conditions [26]. Cells were passaged approximately every 10 days at a ratio of 1:4 (cell culture: new media). Infection of aposymbiotic ISE6 cell lines with *w*albB was carried out using a modified shell vial technique as previously described [27]. Briefly, six replicate glass shell vials (29 × 80 mm) were seeded with ISE6 cells at 80–90% confluency and allowed to adhere to the vial surface for six hours. Then, 500 µL of isolated extracellular *Wolbachia* was added to each shell vial. Shell vials were centrifuged at 2500× *g* for 60 min at 20 °C and the cells transferred to a 25 cm^2^ flask with 5 mL SM and 10% heat-inactivated FBS. In an attempt to increase *Wolbachia* infection rates, the shell vial technique was repeated 3 times. After each transfection, the cells were transferred into a 25 cm^2^ flask containing 5 mL of SM and 10% heat-inactivated FBS. ISE6-*w* cells were passaged every 7–10 days at a ratio of 1:4 (cell culture: new media).

To determine *Wolbachia* infection status post transfection procedures, DNA was extracted from ISE6-*w* cells using Qiagen DNeasy kits (Qiagen, Hilden, Germany) following manufactures instructions and amplified using PCR. PCR for samples consisted of 5 µL of 5× One taq buffer (New England Biolabs, Ipswich, MA, USA) 0.5 µL of 10 mM deoxyribonucleotides triphosphate (dNTP), 0.125 µL One Taq DNA polymerase (1.25 units) (New England Biolabs, Ipswich, MA, USA), 0.5 µL of wspec forward and reverse primers (10 µM) (Supplementary Table S1), 1 µL of (50–60 ng/uL) of isolated DNA, and 13.375 µL of molecular grade water to bring the total reaction volume to 25 µL. A volume of 5 µL of each amplification product was separated on a 1.5% agarose gel, stained with GelRed (Biotium, Hayward, CA, USA), and visualized under ultraviolet illumination.

### 2.2. Fluorescent In Situ Hybridization

Fluorescence in situ hybridization (FISH) was performed on the ISE6 and ISE6-*w* cell lines using a 6-FAM labeled *Wolbachia*-specific probe (Supplementary Table S1) to confirm the presence and absence of *Wolbachia* cells at passage 3 and 14 post *Wolbachia* infection. Cells were grown to 80–90% confluency at 28 °C in 25 cm^2^ flasks, cells were disturbed from the flask by shaking, and 400 µL of the cell suspension was added to an 8-well Nunc Lab-Tek Chamber slide system (Thermo Fisher Scientific, Waltham, MA, USA). FISH procedures were performed on ISE6 and ISE6-*w* as previously described [28]. To visualize ISE6 and ISE6-*w* cells, they were stained with DAPI at room temperature for 5 min followed by three 5 min washes with 1× PBS. The cells were then observed using a Leica TCS SP5 confocal microscope with high-efficiency SP detection, and images were processed using Leica LAS X microscope software (Leica Microsystems, Wetzlar, Germany).

### 2.3. Anaplasma phagocytophilum Propagation, ISE6 Cell Inoculation, and qPCR Quantification

*A. phagocytophilum* was propagated in HL-60 cells in RPMI media supplemented with 10% FBS (Gibco, Waltham, MA, USA) and 1:100 Glutamax (Gibco, Waltham, MA, USA) as previously described [29]. HL-60 cultures were maintained at 37 °C in a humified incubator with 5% CO_2_. The number of infected HL-60 cells needed per flask was estimated using the following formula: number of infected HL-60s × 5 morulae/cell × 19 bacteria/morula × 0.5 (50% recovery) [30]. *A. phagocytophilum*-infected HL-60 cells were centrifuged at 10,000× *g* for 10 min at 4 °C. The supernatant was discarded and the cells were resuspended in ISE6 infection medium (L15C300 supplemented with 5% tryptose phosphate broth (BD, Sparks, MD, USA), 5% heat-inactivated FBS (Gibco, Waltham, MA, USA), 0.1% bovine lipoprotein concentrate (MP Biomedical, Irvine, CA, USA), 25 mM HEPES (Sigma-Aldrich, St. Louis, MO, USA), and 0.25% NaHCO3 (Sigma-Aldrich, St. Louis, MO, USA)). The pH was adjusted to 7.5–7.7 [31]. Bacteria was purified by passing the suspended infected cells through a 27 Ga bent needle. Cell lysates were separated by centrifugation at 1000× *g* for 10 min at 4 °C. The supernatant containing the bacteria was absorbed and inoculated into the ISE6 and ISE6-*w* cells (~5–8 × 10^5^ cells/mL) in 25 cm^2^ flasks. Cells were inoculated with 4.5 × 10^7^ *A. phagocytophilum* cells. Two experimental *A. phagocytophilum* inoculations were completed, one at passage three and the other at passage 14, with one and three biological replicates, respectively. DNA was extracted from ISE6 and ISE6-*w* cells using Qiagen DNeasy kits (Qiagen, Hilden, Germany) following manufactures instructions. *A. phagoytophilum* copy number was determined using qPCR to amplify a fragment of the major surface protein 5 gene (msp5) normalized to actin (Supplementary Table S1) using PowerUp SYBR Green qPCR Master Mix (ThermoFisher, Waltham, MA, USA) and a CFX96 Real-time PCR system (Bio-rad, Hercules, CA, USA) following manufactures instructions. Amplification conditions consisted of 59 °C for 2 m, 95 °C for 10 m, and 40 cycles of 95 °C for 15 s, 59 °C for 1 m. All qPCR were completed as three technical replicates.

### 2.4. Wolbachia Density

*Wolbachia* infection density in ISE6-*w* cells was determined using the same DNA isolations used to quantify *A. phagocytophilum* copy number and qPCR. *Wolbachia* density was determined by amplifying a fragment of the *Wolbachia wsp* gene (Supplementary Table S1) using the same PCR conditions described in the previous section. All reactions were completed in duplicate or triplicate for each DNA sample. The relative abundance of *Wolbachia* in ISE6-*w* cell lines was normalized to actin (Supplementary Table S1).

### 2.5. RNA Isolation and Immune Gene Expression

Qiagen RNeasy mini kits were used to isolate RNA for quantification of host cell immune gene expression. cDNA was synthesized using a Lunascript RT SuperMix Kit (New England Biolabs, Ipswich, MA, USA). qPCR was used to determine host gene expression of p47, relish, JAK, and STAT immune-regulated genes in ISE6 and ISE6-*w* cell cultures (Supplementary Table S1) [7,30]. All amplifications completed at passage 2 were from three biological replicates of cells not inoculated with *A. phagocytophilum*. Amplifications of immune genes at passage 14 were completed from three biological replicates inoculated with *A. phagocytophilum*. All qPCR reactions were performed by amplifying the target immune genes using the same methodology described to determine *A. phagocytophilum* copy number, completed in triplicate, and quantified using the 2^−ΔΔct^ method normalized to actin [30].

### 2.6. Statistical Analysis

Data were checked for significant deviations from normality and equality of variance using a Shapiro–Wilk goodness of fit tests. Differences in *A. phagocytophilum* copy number for the passage 14 experiment were determined using a Kruskal–Wallis multiple comparisons test and followed by Wilcoxon pairwise comparisons for each time point. *Wolbachia* density between passages was compared using an ANOVA and post hoc Bonferroni corrected *t*-tests with an alpha value of 0.008. Differences in immune gene expression in the passage 3 and 14 experiments were determined using an ANOVA and post hoc *t*-tests. All statistical analyses were performed using JMP Pro version 16 (SAS Institute, Cary, NC, USA).

## 3. Results

### 3.1. ISE6 Can Be Transfected with Wolbachia and Impact Anaplasma Transmission

**Figure 1:**
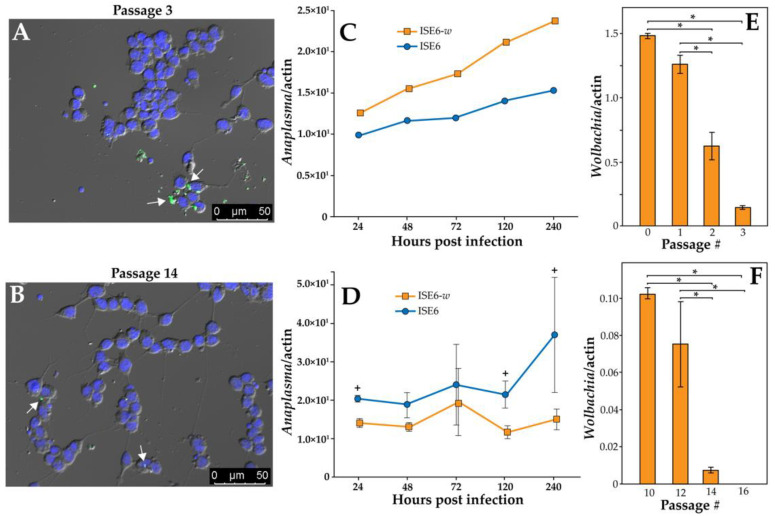
(**A**) Fluorescent in situ hybridization (FISH) staining of ISE6-*w* cells confirming *Wolbachia* infection establishment at passage 3 post transfection, (**B**) FISH staining of ISE6-*w* cells at passage 14, (**C**) *A. phagocytophilum* copy number normalized to the actin gene in ISE6-*w* and ISE6 cells at passage 3, (**D**) *A. phagocytophilum* copy number normalized to the actin gene in ISE6-*w* and ISE6 cells at passage 14. The + above data points represent significant differences according to pairwise Wilcoxon rank sum tests (*p* ≤ 0.05), (**E**) *Wolbachia* copy number in ISE6-*w* cells normalized to the actin gene for passages 0–3 post transfection of *Wolbachia*, and (**F**) *Wolbachia* copy number in ISE6-*w* cells normalized to the actin gene for passages 10–16. The * above the bars represent significant differences according to Bonferroni corrected *t*-tests (*p* ≤ 0.008). All data are represented as mean ± standard error.

Naïve ISE6 cells were transfected with the *w*albB infection after two transfection procedures, as demonstrated by positive PCR tests in passages 0, 1, 2 & 3 post infection and FISH staining (Supplementary Figure S1 and Figure 1A,B). The resulting cell line was named ISE6-*w*. To determine if *Wolbachia* was impacting pathogen replication, we inoculated ISE6 and ISE6-*w* cells at passage 3 post *Wolbachia* infection with *A. phagocytophilum*. *A. phagocytophilum* copy number was observed to be higher in ISE6-*w* when compared to ISE6 cells at all time points up to 240 h post infection (Figure 1C). A similar experiment to examine for effects on *A. phagocytophilum* was repeated with ISE6-*w* and ISE6 cells at passage 14. An overall difference in *A. phagocytophilum* copy number between ISE6 and ISE6-*w* cells was observed when comparing all time points post inoculation (Kruskal–Wallis, chi-squared, 10.65, DF = 1, *p* = 0.001) (Figure 1D). Specifically, differences in *A*. *phagocytophilum* were observed at 24, 120, and 240 h post inoculation when comparing ISE6-*w* and ISE6 cells (Figure 1D). *Wolbachia* density was also measured as a it could be a factor that impacted *A. phagocytophilum* replication and proliferation. *Wolbachia* density decreased significantly when comparing passages 0–3 (ANOVA, F = 47.2, DF = 3, *p* ≤ 0.004) (Figure 1E), suggesting the ISE6-*w* cell line was gradually losing the *w*albB infection. When comparing later passages (10–16) there is evidence of a secondary shift in *Wolbachia* infection density and subsequent loss of infection by passage 16, as determined by an undetectable qPCR and low number of *Wolbachia* cells at passage 14 in FISH images (ANOVA, F = 18.8, DF = 3, *p* ≤ 0.0006) (Figure 1B,F).

### 3.2. Wolbachia Infection in ISE6 Cells Downregulates Immune Gene Expression but Is Dependent upon Wolbachia Density

**Figure 2:**
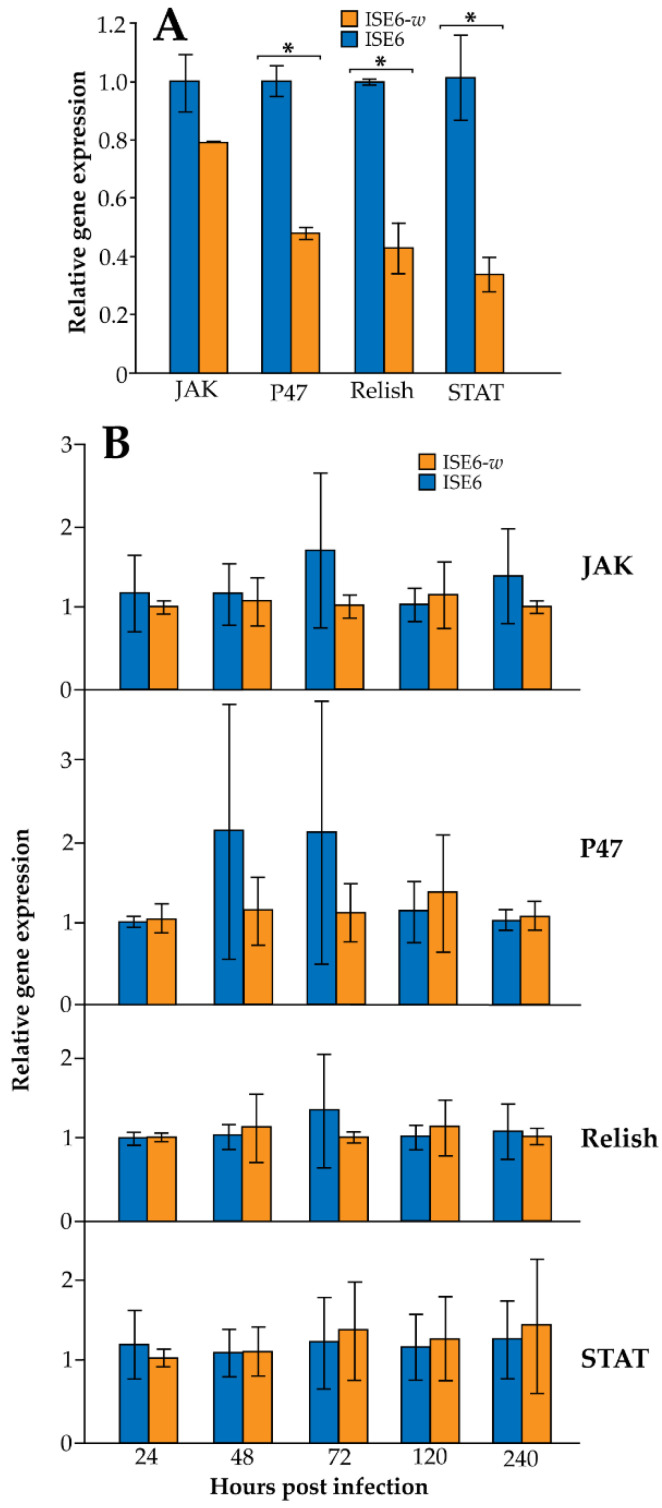
(**A**) Immune response of ISE6-*w* cells after *Wolbachia* infection establishment at passage 3 compared to naïve ISE6 cells. (*) above each bar represent statistical differences according to *t*-tests (*p* ≤ 0.05), (**B**) Immune response of ISE6-*w* and ISE6 cells when inoculated with *A. phagocytophilum* at passage 14. All data are represented as the mean ± standard error.

A significant difference in immune gene expression of ISE6 and ISE6-*w* cells was observed at passage 2 when cells were not inoculated with *A. phagocytophilum* but were infected with *Wolbachia* (ANOVA, F = 42.5, DF = 1, *p* ≤ 0.001) (Figure 2A). No difference in individual gene expression of JAK was observed when comparing ISE6 and ISE6-*w* cells, while a significant downregulation was observed in comparisons of *Wolbachia*-infected and uninfected cells for P47, Relish, and STAT immune genes (*t*-tests, *p* ≤ 0.05) (Figure 2A). Little to no change in immune gene expression was observed when examining for an effect of *Wolbachia* infection status in *A. phagocytophilum* inoculated ISE6 and ISE6-*w* cells (ANOVA, JAK, DF = 1, F = 0.77, *p* = 0.39; P47, DF = 1, F = 0.45, *p* = 0.51; Relish; DF = 1, F = 0.04, *p* = 0.84; STAT, DF = 1, F = 0.02, *p* = 0.89) at passage 14 (Figure 2B). Furthermore, no change in gene expression was observed considering time as an effect post the inoculation of *A. phagocytophilum* (ANOVA, JAK, DF = 1, F = 0.13, *p* = 0.96; P47, DF = 1, F = 0.28, *p* = 0.89; Relish; DF = 1, F = 0.08, *p* = 0.99; STAT, DF = 1, F = 0.11, *p* = 0.98) or when examining for an interaction of *Wolbachia* infection status and time (ANOVA, JAK, DF = 1, F = 0.23, *p* = 0.92; P47, DF = 1, F = 0.29, *p* = 0.88; Relish; DF = 1, F = 0.18, *p* = 0.95; STAT, DF = 1, F = 0.05, *p* = 0.99) at passage 14 (Figure 2B). 

## 4. Discussion

Here, we demonstrate the transfection of ISE6 cells with a *w*albB infection from donor Aa23 mosquito cells. While the intended goal was to generate an ISE6 cell line with a stable *Wolbachia* infection, *w*albB could only infect ISE6 cells for 14 passages. This result agrees with earlier studies that demonstrated that when ISE6 cells were transfected *w*albB and *w*Stri, the infections were only able to persist for 5 and 29 passages, respectively [32]. Taken together, these results suggest a donor *Wolbachia* infection from an arthropod cell line that is not closely related to the receiving arthropod cell line may have difficulty establishing a stable infection, and the host cell range capable of support in vitro *w*albB infections may be limited. It may be necessary to perform additional *Wolbachia* transfection attempts using a *Wolbachia* infection isolated from an arthropod more closely related to ticks to generate a stably infected ISE6 cell line. For example, *Wolbachia* infections isolated from a different tick species or an arachnid may have a greater chance of establishing a long-term *Wolbachia* infection in ISE6 cells in vitro or ticks in vivo. Long-term cultivation of tick cell lines has also been demonstrated to affect genome stability and result in changes in chromosome number genomic changes, which could affect *Wolbachia* transfection stability [33].

In contrast to previous studies, *Wolbachia* in this system does not result in the inhibition of an intracellular pathogen. While most *Wolbachia* pathogen inhibitory studies have focused on viruses, the interaction with intracellular bacteria such as *A. phagocytophilum*, *Wolbachia*, and ISE6 host cells may be exclusive. Previous studies have shown a relatively high (>1 *Wolbachia*/cell) *Wolbachia* density can impact pathogen proliferation and reduce intracellular pathogen replication rates [22,34]. Here, *Wolbachia* density appears to have the opposite effect, and at higher densities results in an increased level of *A. phagocytophilum* proliferation. Unfortunately, because stable *Wolbachia* infections were difficult to maintain in ISE6 cells, we could not replicate experiments examining for an effect of *Wolbachia* infection on *A. phagocytophilum* at passage 3, wherein *Wolbachia* infection densities were higher than in later passages. The immune response in ISE6-*w* cells is also contrary to many previous studies. In ISE6-*w* cells initially transfected with *w*albB, cellular immune response was downregulated. However, this observed immune response was in the absence of *A. phagocytophilum.* Later passages showed a similar immune response between ISE6-*w* and ISE6 cells when inoculated with *A. phagocytophilum*. It is important to note that *Wolbachia* infection density was substantially lower at passage 14 than in earlier passages (1–3) and *Wolbachia* for this reason does not result in any immune response. The opposite trend at passage 14 was observed when *A. phagocytophilum* copy number increased in ISE6 compared to ISE6-*w* cells, but the *Wolbachia* infection was almost lost at this time-point. This observation suggests *A. phagocytophilum* proliferation is reduced in *Wolbachia*-uninfected cells. We are uncertain of why *A. phagocytophilum* copy number was lower in ISE6-*w* cells at passage 14 at some time points compared to the ISE6 cells, but overall cell line health could have been reduced in the ISE6-*w* line harboring the *Wolbachia* infection in earlier passages.

## 5. Conclusions

This work demonstrates short-term, in vitro infections of *Wolbachia* can be established in ISE6 cells and can impact *A. phagocytophilum* proliferation, which suggests the need to examine the interaction of different *Wolbachia* types in tick cells. Ultimately, it would also be important to understand the interactions between the tick host, pathogens, *Wolbachia*, and other microbiota in vivo and the continuing trend of detecting *Wolbachia* infections in natural populations of ticks. Understanding these potentially important interactions may lead to the development of novel tick and tick-borne disease-control approaches.

## Data Availability

Data are available from the corresponding author upon request.

## Supplementary Materials

The following are available online at https://www.mdpi.com/article/10.3390/ijerph19031051/s1, Table S1: Primers used for qPCR to determine *A. phagocytophilum* and *Wolbachia* densities, and immune gene expression. Supplementary Figure S1: PCR amplification results for ISE6-*w* cells post transfection for passages 0–3 and FISH images of ISE6 *Wolbachia* naïve cells.
