# Supplementary material for: Wolbachia Impacts Anaplasma Infection in Ixodes scapularis Tick Cells
Source: Int J Environ Res Public Health. 2022 Jan 18;19(3):1051. doi: 10.3390/ijerph19031051 (PMC8834366; doi:10.3390/ijerph19031051)
Supplement: Supplementary file 1 [file ijerph-19-01051-s001.zip › Supplementary Table S1.pdf]

Supplementary Table S1. Primer sequences used for PCR and qPCR.

Gene [reference]	Forward Sequence	Reverse Sequence
16s (wspec) [1]	5′ – CATACCTATTCGAAGGGATAG – 3′	5′ – AGCTTCGAGTGAAACCAATTC – 3′
JAK [2]	5′ – GAGTACCTGGAAGAGAAG – 3′	5′ – TGTGGATGTAGTAGTAGTG – 3′
Relish [3]	5′ – AGAATGTCCGCCACCGTTTTTTTCTGC – 3′	5′ – CACGTGCACCGCCTCACCATGAAGG – 3′
STAT [2]	5′ – AGGTCAAGGTGTCCATCATC – 3′	5′ – GATACTCCATTGTTCCTGTGTTG – 3′
P47 [3]	5′ – GCCAGGGCCAAGCTTTACC – 3′	5′ – CTTGGACGCTCCAGCGAC – 3′
Actin [3]	5′ – GGTATCGTGCTCGACTC – 3′	5′ – ATCAGGTAGTCGGTCAGG – 3′
Wsp [4]	5′ – CATTGGTGTTGGTGTTGGTG – 3′	5′ – ACCGAAATAACGAGCTCCAG – 3′
Msp5 [5]	5'- TGACACTGTGGTTGAACAAGC -3'	5'- GAAGAAAAGCCGAACATAAGC -3'
Wolbachia 16s [6]	5'-/56-FAM/ AAT CCG GCC GAR CCG ACC C-3'	5'-/56-FAM/ CTT CTG TGA GTA CCG TCA TTA TC-3'

References

1. Werren, J.H.; Windsor, D.M. *Wolbachia* infection frequencies in insects: evidence of a global equilibrium? *Proc Biol Sci* **2000**, *267*, 1277-1285.
2. Liu, L.; Dai, J.; Zhao, Y.O.; Narasimhan, S.; Yang, Y.; Zhang, L.; Fikrig, E. *Ixodes scapularis* JAK-STAT pathway regulates tick antimicrobial peptides, thereby controlling the agent of human granulocytic anaplasmosis. *J Infect Dis* **2012**, *206*.
3. Carroll, E.E.M.; Wang, X.; Shaw, D.K.; O'Neal, A.J.; Chávez, A.S.O.; Brown, L.J.; Boradia, V.M.; Hammond, H.L.; Pedra, J.H.F. p47 licenses activation of the immune deficiency pathway in the tick *Ixodes scapularis*. *Proc. Natl. Acad. Sci. USA* **2018**, *116*, 205–210. https://doi.org/10.1073/pnas.1808905116.
4. Sheehan, K.B.; Martin, M.; Lesser, C.F.; Isberg, R.R.; Newton, I.L. Identification and Characterization of a Candidate Wolbachia pipientis Type IV Effector That Interacts with the Actin Cytoskeleton. MBio 2016, 7.
5. Chávez, A.S.O.; Fairman, J.W.; Felsheim, R.F.; Nelson, C.M.; Herron, M.J.; Higgins, L.; Burkhardt, N.Y.; Oliver, J.D.; Markowski, T.W.; Kurtti, T.J.; et al. An O-Methyltransferase is required for infection of tick cells by *Anaplasma Phagocytophilum*. *PLoS Pathog.* **2015**, *11*, e1005248. https://doi.org/10.1371/journal.ppat.1005248.
6. Rasgon, J.L.; Gamston, C.E.; Ren, X. Survival of *Wolbachia pipientis* in Cell-Free Medium. *Appl. Environ. Microbiol.* **2006**, *72*, 6934–6937. https://doi.org/10.1128/aem.01673-06.
