# Supplementary figures and images for: Wolbachia Impacts Anaplasma Infection in Ixodes scapularis Tick Cells
Source: Int J Environ Res Public Health. 2022 Jan 18;19(3):1051. doi: 10.3390/ijerph19031051 (PMC8834366; doi:10.3390/ijerph19031051)

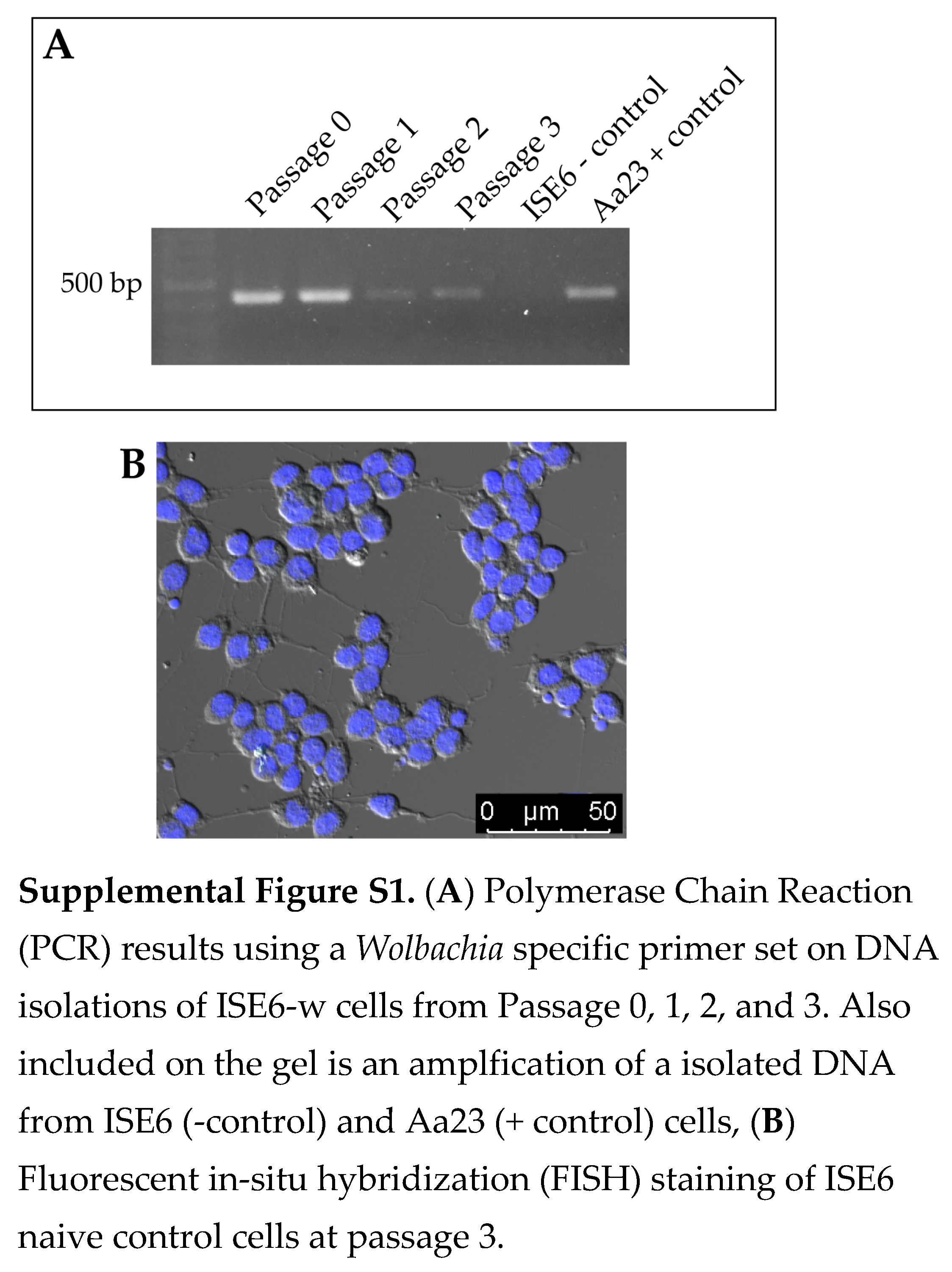

Supplement: Supplementary file 1 [file ijerph-19-01051-s001.zip › Supplemental Figure S1_vs.2.tif]
